# ‘The university should promote health, but not enforce it’: opinions and attitudes about the regulation of sugar-sweetened beverages in a university setting

**DOI:** 10.1186/s12889-017-4626-8

**Published:** 2017-08-01

**Authors:** Elly Howse, Becky Freeman, Jason H. Y. Wu, Kieron Rooney

**Affiliations:** 1 Prevention Research Collaboration, School of Public Health, Sydney Medical School, the University of Sydney, Sydney, Australia; 2 Charles Perkins Centre, the University of Sydney, Sydney, Australia; 3 The George Institute for Global Health, Sydney, Australia; 4 Faculty of Medicine, The University of New South Wales, Sydney, Australia; 5 School of Exercise and Sports Science, Faculty of Health Science, the University of Sydney, Sydney, Australia

**Keywords:** Sugar-sweetened beverages, Young adults, Universities

## Abstract

**Background:**

The study aimed to determine the opinions and attitudes of a university population regarding the regulation of sugar-sweetened beverages in a university setting, primarily looking at differences in opinion between younger adults (under 30 years of age) and older adults (30 years of age or older).

**Methods:**

An online survey was conducted at an Australian university in April–May 2016 using a convenience sample of students and staff between the ages of 16 and 84 years. The survey included questions about consumption of sugar-sweetened beverages and level of agreement and support of proposed sugar-sweetened beverage interventions. Quantitative response data and qualitative open-ended response data were analysed.

**Results:**

Nine hundred thirteen responses from students and staff were analysed. In this population, consumption of sugar-sweetened beverages was low and awareness of the health risks of sugar-sweetened beverages was high. Overall, the surveyed population indicated more support for interventions that require higher levels of personal responsibility. The population did support some environment-centred, population-based interventions, such as increasing access to drinking water and reducing the price of healthier beverage alternatives. However there was less support for more restrictive interventions such as removing sugar-sweetened beverages from sale. Young adults tended to be less supportive of most interventions than older adults.

**Conclusions:**

These findings indicate there is some support for environment-centred, population-based approaches to reduce the availability and appeal of sugar-sweetened beverages in an adult environment such as a university setting. However these results suggest that public health may need to focus less on educating populations about the harms associated with sugar-sweetened beverages. Instead, there should be greater emphasis on explaining to populations and communities why environment-centred approaches relating to the sale and promotion of sugar-sweetened beverages should be prioritised over interventions that simply target personal responsibility and individual behaviours.

**Electronic supplementary material:**

The online version of this article (doi:10.1186/s12889-017-4626-8) contains supplementary material, which is available to authorized users.

## Background

Young adulthood, broadly defined as the life stage occurring after adolescence from the ages of 18 to 30 years of age, is a ‘critical developmental period’ [1]. During this period weight gain is increasingly common, particularly among university students [2]. In Australia, young adults are becoming overweight and obese at a faster rate than other adult age groups [3, 4]. One specific dietary behaviour that has been linked to weight gain, particularly in children and young adults, is the consumption of discretionary foods with added sugars, such as sugar-sweetened beverages (‘SSBs’) [5, 6].

Currently there is focus on implementing population-based approaches to reduce the availability and acceptability of SSBs. These approaches tend to be environment-centred and policy-driven, rather than individual-centred: they aim to change the environment to one of health promoting choices or ‘optimal defaults’ [7]. These approaches require lower levels of personal responsibility or ‘agency’ from individuals to change their behaviour and aim to create environments that readily enable healthier choices [8]. Examples of these approaches include: applying specific taxes or levies to SSB products [9, 10]; removing or restricting access to SSBs in institutional settings such as schools [11, 12]; and changing serving size and placement of SSBs in other settings such as hospitals [13].

Despite the high rates of SSB consumption amongst young adults, few environment-centred, population-based interventions have been aimed at young adult populations. In 2015 the University of California San Francisco phased out SSBs from sale on their university campus, noting their responsibility as a prominent medical university and community leader in helping people to improve their health [14]. To date, no Australian universities have implemented a similar policy. Given that young adults in Australia are the highest adult consumers of added sugars found in SSB products [6] and weight gain is a significant health issue in this population, an important obesity prevention strategy in this population may be to reduce the appeal of and demand for SSBs in post-secondary educational settings like universities.

Other studies of university populations in Australia have looked at food preferences and purchasing behaviours [15] as well as soft drink consumption [16, 17]. This study assessed the opinions, attitudes, and knowledge of a university population in regards to the health risks of SSBs as well as the regulation of the sale and promotion of these products on a university campus. The study also aimed to determine the level of acceptability or support for a number of proposed SSB-related interventions including removing SSBs from sale. The hypothesis was that young adults would be higher consumers of SSBs and would be less likely than older adults to support interventions on campus to reduce the availability and acceptability of SSBs.

## Methods

### Study design

An online survey was developed, tested and agreed on by academic staff and research students who are part of a population-based health promotion initiative at the University of Sydney, Australia [18]. The survey was comprised of forty-one questions in total [Additional file 1]. The survey was informed by previous food environment research at the University of Sydney [15, 19].

In this survey, sugar-sweetened beverages were defined as pre-packaged beverages that included any form of sugar added during the manufacturing process. A list of beverages were provided as an example, which included soft drinks (‘sodas’), fruit juices, energy drinks, sports drinks, sugar-added waters, and flavoured milk. Alcoholic beverages, plain (unflavoured) milk products, 100% fruit juices (with no added sugar), and tea and coffee were excluded from this definition.

### Setting and participants

The University of Sydney is a research-intensive higher education institution of approximately 57,000 students and 8000 staff, with the largest campus based in the inner city suburbs of Sydney, New South Wales, Australia. 85% of students at the University of Sydney are aged under 30 years and 57% are female. Approximately 59% are enrolled in an undergraduate degree (Bachelor-level) and 36% are enrolled in a science, health or medical faculty.

Data was collected over a four week period 4 April 2016–2 May 2016 through a convenience sample of university staff and students, who were invited to participate via email, flyers, posters, electronic newsletters and social media. The survey was approved by the Human Research Ethics Committee of the University of Sydney, project number 2016/124.

A total of 954 survey responses were received. However, 41 responses did not consent to participating or left the survey blank and were removed from analysis. A total of 913 responses were included in the final analysis.

### Independent variables

The independent variable of interest was age, collected as a continuous variable and analysed as a dichotomous categorical variable (< 30 years, ≥ 30 years). The survey also captured other demographic variables, such as gender, faculty and primary role.

### Outcome variables

#### SSB consumption

SSB consumption was measured in three ways. Regular SSB consumption was self-reported using a five interval scale ranging from ‘At least once a day’ to ‘I do not consume SSBs’. This was recoded as a dichotomous variable (‘weekly or more often’; ‘less often or not at all’). Recent consumption was measured continuously (number of occasions of SSBs consumed in last 7 days); this was also recoded as a dichotomous variable (‘Zero’; ‘One or more’). Participants were asked the size of the last SSB consumed using a seven interval scale ranging from ‘I do not drink SSBs’, ‘less than 250 mL’ to ‘1 Litre bottle’. This was recoded as dichotomous (‘Don’t drink SSBs or less than 250 mL’; ‘Greater than 250 mL’).

#### Opinions and knowledge about the health impacts of SSB consumption

Opinions about the health impacts of consuming SSBs were asked using interval scales which were recoded into dichotomous variables. Knowledge about the health impacts of consuming SSBs were asked using a ‘tick box’ approach, with the offered answers ‘Obesity and overweight’, ‘Type 2 diabetes’, ‘Cardiovascular disease’, ‘Metabolic syndrome’, ‘Stroke’ and ‘Other’.

#### Opinions and attitudes towards the university environment

Opinions and attitudes were measured using a five degree Likert scale in response to a series of statements about the university environment. The five possible responses were ‘Strongly agree’, ‘Agree’, ‘Neither agree nor disagree’, ‘Disagree’, and ‘Strongly Disagree’. During analysis these responses were recoded as dichotomous outcomes: ‘Agree’ and ‘Neutral/Disagree’ (which included ‘Neither agree nor disagree’).

#### Opinions and attitudes regarding proposed SSB interventions

A five degree Likert scale was also used to measure support for sixteen proposed regulatory SSB interventions on campus. These five responses were ‘Extremely supportive’, ‘Somewhat supportive’, ‘Neutral’, ‘Somewhat unsupportive’ and ‘Extremely unsupportive’. During analysis these responses were recoded as dichotomous outcomes: ‘Supportive’ and ‘Not supportive’. ‘Neutral’ responses were coded as negative responses (the reference category), as the purpose of the study was to look at outright levels of agreement and support for particular statements and proposed interventions.

### Statistical methods

Statistical data analyses were performed using IBM SPSS version 22.0. Pearson’s Chi-square test was used to examine associations between age (categorical) and the outcome variables of interest (SSB consumption; opinions about health risks of SSBs; opinions about the university environment; and opinions about SSB interventions). An independent samples t-test was used to examine differences in the mean SSB consumption between young adults and older adults.

### Content analysis

Content analysis was conducted by two of the researchers (EH and BF) to analyse the results of the open-ended questions, 321 responses in total [Additional file 2]. A third of survey participants provided a brief comment or longer paragraph of feedback at the end of the survey. A process of emergent coding was used whereby the first forty responses were coded together by authors EH and BF. Fifteen themes were identified through this inductive process, with thirteen of the themes related directly to SSB interventions. The remaining two themes accounted for unclear responses and responses referring to the survey questions or issues not raised in the survey, for example referring to views on alcohol. Many responses contained multiple themes, with a total of 617 statements across the 321 responses included in the analysis. The responses were each coded individually by EH and BF. The two sets of coding were cross-checked and any inconsistencies were resolved by the researchers.

## Results

### Participants

The mean age of participants was 33.1 years (± 13.0 years) and the age range was 16 to 84 years (see Table 1). The sample contained similar proportions of young adults (<30 years of age, 50.5%) and older adults (≥ 30 years of age, 49.5%). More women (69.2%) than men (29.5%) participated in the survey. Slightly more students (52.4%) than staff (47.6%) participated. The students who participated tended to be undergraduate (57.7% of student participants), enrolled full time (87.7% of students), and an Australian or New Zealand citizen or permanent resident (90.2% of students). Staff who participated tended to be professional or general staff (69.4% of staff participants) and employed full time (70.4%). Over half of all participants (53.8%) came from a science, medical or health faculty.

**Table 1:** Survey participant characteristics (*n* = 913)

Survey participant characteristics	mean(SD) or n (%)^1^
Age (years)	33.1 (13.0)
Age (group)	
Young adults (<30 years)	461 (50.5)
Older adults (= > 30 years)	452 (49.5)
Gender	
Female	632 (69.2)
Male	269 (29.5)
Other/did not wish to provide this information	12 (1.4)
Primary university role	
Student – undergraduate	276 (30.2)
Student – postgraduate (coursework and research)	202 (22.1)
Staff – Academic	105 (11.5)
Staff – Professional/general/other	330 (36.2)
Student status	
Full time enrolment	415 (87.7)
Part time enrolment	58 (12.3)
Student country	
Domestic (Australian or NZ citizen or resident)	431 (90.2)
International (student visa)	47 (9.8)
Staff status	
Full time	300 (70.4)
Part time	68 (16.0)
Casual/contract/other	58 (13.6)
Faculty	
Science, health or medical faculty^a^	491 (53.8)
Other faculty or unit^b^	422 (46.2)

^a^includes: science, agriculture, veterinary science, medicine, nursing, pharmacy, dentistry, health sciences

^b^includes: arts, education, business, law, engineering, architecture, music, fine arts

^1^All variables were presented as n(%) except age (years) which was presented as mean(SD)

### SSB consumption

Self-reported SSB consumption was low in this population. Almost three quarters of participants reported they usually drink SSBs less than once a week or not at all (*n* = 656, 73.4%, see Table 2). However, there were discrepancies in this self-reporting, as when participants were asked about SSB consumption in the last 7 days, more than half of participants (*n* = 473, 52.9%) reported consuming SSBs on one or more occasions. Over half of participants (*n* = 456, 53.1%) reported the size of the last SSB consumed was 250 mL or less (including those who indicated they do not consume SSBs). All three outcome variables related to consumption indicated a significant relationship between age and self-reported consumption of SSBs, suggesting that a greater proportion of young adults than older adults reported consuming SSBs, although the level of association was moderate. There was also a significant difference in the mean number of occasions of SSB consumed in the last seven days for younger adults (m = 2.02, SD = 3.87) compared to older adults (m = 1.02, SD = 2.04), t(892) = 4.837, *p* < 0.001, 95% CI 0.595 to 1.408.

**Table 2:** Associations between SSB consumption characteristics and age

SSB consumption	<30 years n (%)	≥30 years n (%)	Total n (%)	Χ^2^ (df)	Cramer’s V	p*
How regularly do you consume SSBs?						
Less than once a week or not at all	284 (31.8)	372 (41.6)	656 (73.4)	48.893 (1)	0.234	**<0.001**
Once a week or more often	166 (18.6)	72 (8.1)	238 (26.6)			
What was the size of the last SSB you consumed?						
250 mL or less/do not consume SSBs	194 (22.6)	262 (30.5)	456 (53.1)	22.639 (1)	0.162	**<0.001**
Larger than 250 mL	237 (27.6)	166 (19.3)	403 (46.9)			
In the last week, on how many occasions did you consume at least 1 SSB?						
Zero occasions	164 (18.3)	257 (28.7)	421 (47.1)	41.226 (1)	0.215	**<0.001**
1 or more occasions	286 (32.0)	187 (20.9)	473 (52.9)			
	mean (SD)	mean (SD)		t (df)	95% CI	p #
In the last week, on how many occasions did you consume at least 1 SSB?	2.02 (3.87)	1.02 (2.04)		4.837 (892)		**<0.001**

*Chi-square tests of proportion used to compare SSB consumption characteristics according to age group

# Independent samples t-test used to compare occasions of SSB consumption in last 7 days according to age group

*P*: Bolded indicates significant associations

### Opinions and knowledge about the health impact of SSB consumption

Most participants agreed that the consumption of SSBs increased the risk of ill health (*n* = 749, 94.8%) (see Table 3). There was no difference in agreement between age groups. When asked if they would consider choosing a diet SSB for health reasons, fewer than half of the participants (*n* = 334, 42.3%) agreed. A higher proportion of young adults agreed with this statement compared to older adults (24.1 and 18.2% respectively, *p* = 0.003), however the level of association was weak.

**Table 3:** Associations between opinions and knowledge about the health impacts of SSBs and age

Questions and statements about health impacts of SSBs	<30 years n (%)	≥30 years n (%)	Total n (%)	Χ^2^ (df)	Cramer’s V	*p*
Do you believe that consumption of SSBs increases the risk of ill health?						
Yes	379 (94.5)	370 (95.1)	749 (94.8)	0.145 (1)	0.014	0.703
No/not sure	22 (5.5)	19 (4.9)	41 (5.2)			
I would consider choosing a diet or zero SSB for health reasons						
Agree, I would choose a diet SSB	190 (24.1)	144 (18.2)	334 (42.3)	8.690 (1)	0.105	**0.003**
Disagree, I would not choose diet SSBs	211 (26.7)	245 (31.0)	456 (57.7)			

Chi-square tests of proportion used to compare opinions and knowledge about health impact of SSBs according to age group

*P*: bolded indicates significant associations

**Fig. 1:**
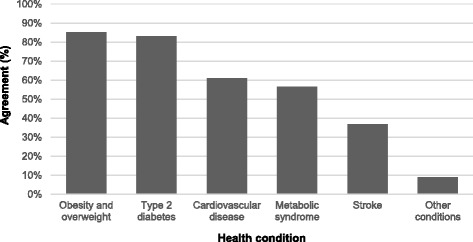
Percentage of agreement of health conditions associated with SSB consumption

Participants also agreed that consuming SSBs was associated with a number of health conditions (see Fig. 1). Participants agreed that SSBs increased the risk of: obesity and overweight (*n* = 777, 85.1% of cases); Type 2 diabetes (*n* = 758, 83.0% of cases); cardiovascular disease (*n* = 558, 61.1%); metabolic syndrome (*n* = 517, 56.6%), stroke (*n* = 335, 36.7%), with a smaller number of respondents (<10%) also identifying links between SSBs and a range of other conditions, the most common of which were dental problems (such as caries, toothy decay and gum disease).

### Opinions and attitudes towards the university environment

There was a high level of agreement amongst participants regarding whether the university should promote health (*n* = 745, 94.5%), whether the university should promote healthier products (*n* = 708, 89.8%) and whether products such as SSBs were harmful for the environment (*n* = 565, 71.7%). A majority of participants also agreed that the university should regulate the sale (*n* = 493, 62.6%) and promotion (*n* = 632, 80.2%) of unhealthy products, however there were some differences in proportion of agreement and disagreement between the two age groups. Younger adults were significantly less likely than older adults to agree with the university regulating the sale and promotion of unhealthy products on campus, however the level of association was weak (see Table 4).

**Table 4:** Opinions about the university environment (*n* = 913) by age group

Statements about the university environment	<30 years n (%)	≥30 years n (%)	Total n (%)	Χ^2^ (df)	Cramer’s V	*p* value
I believe the university should promote the health of its students and staff						
Agree	372 (47.2)	373 (47.3)	745 (94.8)	2.689 (1)	0.058	0.101
Disagree	27 (3.4)	16 (2.0)	43 (5.5)			
I believe the university should regulate the sale of unhealthy products on its campuses						
Agree	230 (29.2)	263 (33.4)	493 (62.6)	8.351 (1)	0.103	**0.004**
Disagree	169 (21.4)	126 (16.0)	295 (37.4)			
I believe the university should regulate the promotion of unhealthy products on its campuses.						
Agree	307 (39.0)	325 (41.2)	632 (80.2)	5.412 (1)	0.083	**0.020**
Disagree	92 (11.7)	64 (8.1)	156 (19.8)			
I believe the university should promote healthier products.						
Agree	349 (44.3)	359 (45.6)	708 (89.8)	5.015 (1)	0.080	**0.025**
Disagree	50 (6.3)	30 (3.8)	80 (10.2)			
I believe that products such as SSBs are harmful for the environment.						
Agree	275 (34.9)	290 (36.8)	565 (71.7)	3.075 (1)	0.062	0.080
Disagree	124 (15.7)	99 (12.6)	223 (28.3)			

Chi-square tests of proportion used to compare opinions about the university environment according to age group

*P*: bolded indicates significant associations

### Opinions and attitudes regarding proposed SSB interventions on campus

The interventions with the greatest levels of support (≥ 75% support) tended to be environment-centred interventions involving some change to the retail or physical environment but still requiring a certain degree of choice or agency by individuals to make healthier decisions, such as: lowering the price of and promoting the placement of healthier alternative beverages; increasing access to free drinking water; and adding nutritional information to fridges and vending machines (see Table 5).

**Table 5:** Levels of overall support for SSB interventions by type of intervention and age

Proposed SSB intervention	<30 years support (%)	≥30 years support (%)	Total support (%)	Cramer’s V	*p* value
Product					
Increase access to free drinking water	95.7	97.9	96.8	0.062	0.084
Limit serving size of SSBs to 250 mL	54.3	65.7	59.9	0.117	**0.001**
Replace SSBs with water products	46.5	55.8	51.0	0.093	**0.010**
Remove SSBs from vending machines	37.2	51.6	44.2	0.145	**<0.001**
Remove SSBs from all campus outlets and vending machines	31.7	42.9	37.2	0.117	**0.001**
Provide access to SSBs only in bars	35.7	34.8	35.3	0.009	0.801
Replace SSBs with diet or low sugar versions	35.7	32.7	34.2	0.031	0.384
Pricing					
Lower price of water and diet beverages	80.4	73.0	76.8	0.087	**0.015**
Increase price of SSBs and reinvest money in healthier activities	64.8	70.7	67.7	0.063	0.080
Remove discounts on SSBs	51.8	68.6	60.0	0.172	**<0.001**
Increase price of SSBs	48.7	55.5	52.1	0.068	0.059
Placement					
Encourage placement of healthier beverages in fridges and vending machines	86.4	91.4	88.8	0.078	**0.029**
Remove SSBs from display	39.4	41.9	40.6	0.025	0.488
Information, education and marketing					
Add nutritional information to fridges and vending machines	84.2	84.0	84.1	0.002	0.958
Run a social marketing campaign to educate staff and students about SSBs	70.6	79.3	74.9	0.100	**0.005**
Remove SSB sponsorship and promotions	61.3	77.0	69.0	0.169	**<0.001**

Chi-square tests of proportion used to compare levels of overall support for SSB interventions by age group

*P*: bolded indicates significant associations

The least supported interventions (< 50% total support) were those which suggested some form of removal or restriction of SSBs in outlets and vending machines as ‘default’ changes to the environment; these changes require less individual responsibility and agency for people to make healthier choices. The least supported intervention was not the proposal to remove SSBs completely from campus, but the proposal to replace SSBs with diet or zero sugar SSBs (34.2% support overall).

Young adults were generally less supportive than older adults of the proposed interventions. There was a significant difference in the level of support demonstrated by young adults for interventions such as: limiting the serving size of SSBs; and removing SSBs from all outlets and vending machines. Young adults also demonstrated less support for removing discounts on SSBs and removing SSB sponsorship and promotions. For these interventions, the strength of the association was moderate.

### Further comments about SSBs on campus

There were two themes that consistently occurred (111 times each) in the open-ended responses. One theme was the belief that a university campus is a population of adults who should be free to make their own decisions or choices. The other theme was that healthier beverage options should be more available on campus.

Other consistently-occurring themes included: the belief or view that educating staff and students on health, including beverage choice, is appropriate and effective (72 in total); banning SSBs from campus will be ineffective (71 in total); and health concerns about the consumption of diet or sugar-free SSBs (56 in total). A smaller number of respondents referred to other issues such as the need for or enjoyment of SSBs (38 in total), and comments about the broader campus food environment being unhealthy (35 in total).

### Adults should be free to make their own decisions or choices

About 10% of respondents indicated they felt strongly about being able to choose to purchase particular types of beverages on campus. Common phrases included the terms ‘personal responsibility’, ‘autonomy’, ‘informed’, ‘individual choice’, ‘free choices’, and ‘right’, for example:

*“I think people have the right to make their own choices (even if they’re not always good ones)”*

Many of these respondents noted they felt it was inappropriate for adults to be treated like children:

*“People should make informed decisions but treating them like 5 year olds is ridiculous”*

The perceived infantilization of adults reflects Hoek [20]’s point about a ‘nanny state’ that ‘evokes unattractive connotations by casting adults as children who can neither exert appropriate personal autonomy nor make their own decisions.’ It is interesting that a small number of respondents (13 in total) used the exact terminology of the ‘nanny state’, for example:

*“The nanny state does not need to extend to the uni campus.”*

These attitudes also suggest some resistance to ‘engineering’ the campus environment to influence people’s choices, indicating this population may be more supportive of individual-centred interventions and approaches to SSBs:

*“I don’t believe in regulation, people should be supported to make their own informed choices.”*

### Make healthy beverage options more available

The other main theme expressed by respondents relates to the provision of healthier beverage options on campus. Respondents felt that if healthier options were available, affordable and promoted, then staff and students would choose to buy them:

*“actively promoting healthy options and making them readily and cheaply available should be our aim.”*

Some of the strategies suggested including lowering the cost for healthier options as an incentive:

*“there should be more incentive to choose healthier options (e.g. price drop)”*

Respondents who referenced this theme also referred to increasing the availability of tap water on campus:

*“There need to be more places to fill up water bottles on campus -there should be no need to pay for water.”*

These comments suggest staff and students are aware of the need to ensure that particular interventions (such as phasing out certain SSB products or increasing the price) need to be accompanied by other interventions that increase the availability of healthier options, for example providing more water fountains on campus.

### Other themes

Survey respondents also felt that educating the population on the health risks of SSBs was acceptable, particularly in the context of education or information that helps consumers make the ‘right’ choice. Another important theme referenced concerns about diet or sugar-free SSBs in terms of impact on physical and dental health, including references to cancer and potentially harmful additives such aspartame.

## Discussion

This study is the first of its kind to survey a university population about attitudes towards SSB interventions on campus. The results reflected trends found in other surveys and population data in Australia, namely that young adults are higher consumers of SSBs than older adults [3, 6]. While results indicated a high level of awareness of the health impacts associated with SSB products, there was some resistance to SSB regulation, particularly environment-centred regulations involving ‘optimal defaults’. This is in line with the findings of other Australian research: that there is community support for more individual-centred initiatives (such as nutritional labelling) but less support for restricting promotions and taxing or increasing the price of unhealthy foods [21].

These results suggest there is a challenge for public health in balancing the philosophical positions of ‘individual’ and ‘environment’, particularly in specific settings like universities. To address major health issues such as obesity requires ‘strategies that integrate these two philosophical positions’ [7]. Although it should be noted that the participants in this study agreed with statements around the university promoting health and restricting the sale and promotion of products associated with harm, the responses to the proposed interventions combined with the commentary data suggest that at present many of the environment-centred interventions around SSBs are less acceptable to highly educated populations who believe strongly in notions of individual responsibility and freedom of choice. This seems to be especially the case for the young adult participants. The results suggest the need to capitalise on support for individual-centred interventions or approaches while building support over the longer term for environment-centred, population-based measures. The latter approaches, particularly those that are multicomponent, may have a greater impact in addressing obesity [22].

### Young adults less supportive of regulatory approaches than older adults

Half of the proposed SSB interventions had moderately lower levels of support amongst young adults when compared to older adults, however this may reflect that the young adults in this sample also tended to report higher levels of SSB consumption. Environment-centred interventions such as pricing strategies and removal of discounts were particularly unpopular amongst young adults, who are more likely to have discount cards and are sensitive to the pricing of foods and beverages [15, 22]. Young adults were also less supportive of measures to remove the sponsorship and promotion of SSBs at student events. This is concerning as young adults are a crucial target group of SSB companies, who use a range of sophisticated marketing measures including social media to promote and normalise SSBs consumption [23]. Recent marketing approaches in Australia include a ‘guerrilla’ campaign on university campuses by an SSB company seeking to ‘infiltrate’ orientation periods and events [24]. Universities in the US have also been criticised for allowing significant levels of SSB promotion to young adults on their campuses [25]. The results of this survey suggest there is a need for young adults to be included in campaigns around interventions to reduce discretionary food and beverage products. Given that 69% of this sample (and 61% of young adults in this sample) supported removing the sponsorship and promotion of SSBs products, universities should consider as a priority and acceptable intervention the restriction or phasing out of direct marketing of SSBs on their campuses, particularly during orientation weeks.

### Individual-centred interventions more acceptable than ‘optimal defaults’

Proposed interventions that were individual-centred or reliant on higher levels of personal responsibility and engagement were more likely to have a higher level of support. There was much less support for interventions that aimed to change the environment to make individuals’ default response healthier, such as removing SSB products entirely or removing them from display. Given the proposed interventions touched on issues of personal versus environmental responsibility, it is not surprising that that some of the participants specifically referenced it was not the ‘role’ of the university to determine access to particular products. These comments frequently conceptualised university as the opposite of school – that a university is a population of adults in comparison to a population of children, where adults ‘know better’ and children don’t (or are unable to). This may reflect the view that certain regulations or ‘optimal default’ changes to the food and beverage environment are acceptable for children (for example, removing junk food advertising during children’s television shows, or removing certain products from school canteens) but are not acceptable for adults. This suggests that even in an educated university population, many believe that adults should be able to take full personal responsibility for their food and beverage choices and change their behaviour.

However, these views about personal responsibility ignore the powerful environmental cues at play which reduce the opportunity for people to make independent and ‘free’ choices [26]. The question of personal responsibility is therefore a distraction, given most environments including university campuses already have as their ‘default’ choice the sale and promotion of energy-dense, nutrient-poor foods and beverages. While the sample was highly educated about the health risks of SSBs, participants did seem naïve about the concept of ‘free choice’ on campus, not recognising that the campus environment is already manipulated by exclusive sales agreements designed to heavily impact consumer choice for the benefit of external commercial interests (such as major multinational beverage companies). Although almost 95% of participants agreed with the statement “*I believe the university should promote the health of its students and staff”*, the widely-accepted and commonly-expressed belief of personal responsibility, particularly in an educated population, suggests a clear challenge for implementing environment-centred interventions in primarily adult settings.

### Concerns around replacement products

Participants in this survey also expressed concern around the perceived health and environmental impact of alternative products to SSBs, such as diet or artificially-sweetened SSBs and plastic bottled beverages (including water products). It is interesting there was some evidence in this sample that younger adults may more supportive than older adults of measures to replace full sugar drinks with diet SSBs and may agree with choosing diet SSBs for health reasons. However there is much less available evidence about the possible health risks of these products as well as ongoing concerns about their environmental impact [27]. There is also a lack of evidence regarding whether replacing SSBs with diet drinks will have any impact on obesity or weight gain [28]. Gathering further evidence about replacement or alternative products is therefore crucial, as the most acceptable interventions for retailers seem to be those which do not impact on revenue: replacing full sugar drinks with diet versions; reformulating products with alternative ingredients; or the discounting of diet and water products. However if these interventions are not acceptable to consumers, pose a risk to the health of people and environment, or have no impact on obesity levels, policymakers and public health professionals may need to consider alternative interventions to reduce SSB consumption.

### Limitations

As a convenience sample was used, the response rate was low (approximately 1.5% of the population in question). As a result, those who participated were self-selected and may not represent the broader population at large. For example, there was an over-representation of female participants, staff participants, and participants coming from a science, health or medical faculty. This has the potential to bias the findings, as it may be that female students and those from a medical or health faculty are more likely to be health-conscious and thus have predisposed ideas about SSBs, diet and impact on health. However, this survey was not aiming to be representative: it aimed to serve as an initial ‘snapshot’ of perceptions and attitudes about SSBs in general on a specific university campus. As such the results may not reflect broader attitudes, although they are a useful starting point from which to develop further research and trial interventions in a highly educated, adult population such as a university population.

## Conclusions

There is growing interest in environment-centred, population-based approaches to reduce the availability and acceptability of SSBs amongst both children and adults, due to the links between SSB consumption, obesity, chronic disease and poor dental health. Products such as SSBs are heavily and aggressively marketed and promoted, particularly to young adults. Yet, given that knowledge of associated health effects are high, education campaigns on SSBs seem to be missing the point. A more effective and useful campaign could be one that directly engages with key groups and challenges concepts such as ‘free choice’ about the food and beverage environment while teaching skills of critical analysis when it comes to marketing.

The information from this survey suggests there are a range of interventions that universities can implement. One strategy could be to combine a number of interventions over time while gradually increasing the acceptability of environment-centred, population-based interventions – as what occurred with tobacco reform. However, as indicated by the results of the survey, any measure or intervention needs to be communicated effectively to the population in question, and utilise existing support for institutions and communities to promote the health of their people.

The question of why young adults in this sample were less supportive of environment-centred interventions warrants further investigation. Follow up research could explore the values and beliefs that young adults (including those from a wider variety of backgrounds beyond university students) hold around the normalisation of food and beverage products including SSBs and their availability and promotion in everyday settings. This could better inform public health strategies for reducing SSB consumption and improving diet amongst young adults while addressing an important risk factor for obesity and poor health in this population.

## Additional files

Additional file 1: Copy of study survey. (PDF 351 kb)

Additional file 2: Thematic category descriptors for study qualitative data. (DOCX 20 kb)
