# Supplementary material for: ‘The university should promote health, but not enforce it’: opinions and attitudes about the regulation of sugar-sweetened beverages in a university setting
Source: BMC Public Health. 2017 Aug 1;18:76. doi: 10.1186/s12889-017-4626-8 (PMC5540300; doi:10.1186/s12889-017-4626-8)
Supplement: Supplementary file 1 — Copy of study survey. (PDF 351 kb) [file 12889_2017_4626_MOESM1_ESM.pdf]

Sugar-Sweetened Beverages and their Sale and Promotion in a University

1. Introduction and Participant Information Statement

You are invited to take part in a research study about exploring the perceptions and attitudes of university students and staff towards sugar-sweetened beverages and their regulation in a university setting.

You have been invited to participate in this study because you are a student and/or staff member of the University of Sydney and the research generated through this study may be of benefit to your health and wellbeing. Your participation in this study may benefit the University of Sydney community by providing feedback and research on creating a healthier food and beverage environment on campus. This Participant Information Statement tells you about the research study. Knowing what is involved will help you decide if you want to take part in the research. Please read this information carefully and ask questions about anything that you don't understand or want to know more about.

Participation in this research study is voluntary.

By giving your consent to take part in this study you are telling us that you:

- Understand what you have read.
- Agree to take part in the research study as outlined below.
- Agree to the use of your personal information as described.

A copy of this Participant Information Statement is available byclicking here or pasting this URL into your browser: http://sydney.edu.au/dam/corporate/documents/about-us/values-and-visions/HSU%20Participant%20Information%20Statement.pdf.

This study is funded by Healthy Sydney University, an initiative of the University of Sydney that promotes the health and wellbeing of University of Sydney staff and students.

The ethical aspects of this study have been approved by theHuman Research Ethics Committee of the University of Sydney, Project Number 2016/124.

* 1. I have read the Participation Information Statement. You must answer 'Yes' if you wish to participate in the study. If you answer 'No' you will not be able to participate further in the study.

- ◯ Yes, I have read the Participation Information Statement.
- ◯ No, I have not read the Participation Information Statement.

2. Participant Consent Form

Study Name: Perceptions and Attitudes of University Students and Staff Regarding Sugar-Sweetened Beverages and Their Sale and Promotion in a University Setting

I agree to take part in this research study.

In giving my consent I state that:

- I understand the purpose of the study, what I will be asked to do, and any risks/benefits involved.
- I have read the Participant Information Statement and have been able to discuss my involvement in the study with the researchers if I wished to do so.
- The researchers have answered any questions that I had about the study and I am happy with the answers.
- I understand that being in this study is completely voluntary and I do not have to take part. My decision whether to be in the study will not affect my relationship with the researchers or anyone else at the University of Sydney now or in the future.
- I understand that I can withdraw from the study at any time by not completing or submitting the survey, or at any time by emailing the investigators to remove my survey at any point.
- I understand that personal information about me that is collected over the course of this project will be stored securely and will only be used for purposes that I have agreed to. I understand that information about me will only be told to others with my permission, except as required by law.
- I understand that the results of this study may be published, and that publications will not contain my name or any identifiable information about me.

* 2. Do you consent to the above terms? By answering 'Yes', you consent that you have read the Participant Information Statement and are willing to answer the questions in this survey. If you answer 'No', you will not be able to participate in the survey.

- ○ Yes, I wish to participate in this study.
- ○ No, I do not wish to participate in this study.

3. Demographic Information

* 3. What is your gender?

- ◯ Female
- ◯ Male
- ◯ Other
- ◯ I do not wish to provide this information.

* 4. What is your age in years?

[ ]

* 5. What is the level of the highest qualification that you have completed?

- ◯ PhD or Doctorate
- ◯ Masters degree (research or coursework)
- ◯ Bachelor degree
- ◯ Certificate or Diploma (including vocational training)
- ◯ Final year of high school (HSC; Leaving Certificate)
- ◯ Primary School
- ◯ Other (please specify)

[ ]

* 6. What is your primary role at the University of Sydney? Please choose the option that best describes you at this point in time.

- ◯ Student - undergraduate (eg. Bachelor degree, including Honours)
- ◯ Student - postgraduate coursework (eg. Masters; Juris Doctor).
- ◯ Student - postgraduate research (eg. PhD; M Philosophy or Research)
- ◯ Staff - academic
- ◯ Staff - professional/general
- ◯ Affiliate - Honorary, contractor or consultant
- ◯ Other (please specify)

[ ]

* 7. What faculty or unit are you primarily associated with?

Sugar-Sweetened Beverages and their Sale and Promotion in a University

4. Student Information

* 8. What is your current student status?

- ◯ Full time enrolment
- ◯ Part time enrolment
- ◯ Other (please specify)

* 9. Are you a domestic or international student?

- ◯ Domestic (Australian or New Zealand citizen or permanent resident)
- ◯ International (student visa)

5. Staff Information

* 10. In what capacity are you employed by the University of Sydney?

- ◯ Full time
- ◯ Part time
- ◯ Casual
- ◯ Contract
- ◯ Other (please specify)

[ ]

6. Questions about Sugar-Sweetened Beverages

For the purposes of this study, sugar sweetened beverages ('SSBs') are defined as pre-packaged beverages that include any form of sugar added during the manufacturing process.

According to the Australian Dietary Guidelines, the Rethink Sugary Drink campaign and the World Health Organisation, these products include:
- Soft drinks eg. Coke, Pepsi, Lemonade (Please note this does not include diet or artificially sweetened drinks)
- Fruit juice
- Sweetened iced tea drinks
- Energy drinks eg. V, Redbull, Mother (Please note this does not include diet or artificially sweetened drinks)
- Vitamin waters
- Sports drinks eg. Powerade, Gatorade
- Flavoured milk (including iced coffee and yoghurt drinks)
- Liquid breakfast drinks eg. Up & Go.

For the purposes of this study, sugar-sweetened beverages does not include alcoholic beverages, tea and coffee where sugar is added by the consumer, 100% fruit juices, and plain (unflavoured) milk products.

* 11. How regularly do you consume sugar-sweetened beverages? Please choose the answer that best applies to you.

○ At least once a day

○ At least once or twice a week

○ Several times a month

○ Several times a year

○ I do not consume sugar-sweetened beverages.

* 12. In the last week, on how many occasions did you consume at least one sugar sweetened beverage?

[ ]

* 13. What was the size of the last sugar-sweetened beverage you consumed?

- ◯ Less than 250mL (standard size glass)
- ◯ 250mL-400mL (a regular can of soft drink is 375mL)
- ◯ 400mL-600mL
- ◯ 600mL-800mL (a regular bottle of soft drink is 600mL)
- ◯ 800mL-1000mL
- ◯ 1 Litre bottle
- ◯ I don't know
- ◯ I do not drink sugar-sweetened beverages.

* 14. In a usual week, on how many occasions do you think most (at least 51%) of your friends, peers, colleagues and/or family would consume a sugar-sweetened beverage?

____

* 15. Where do you usually purchase sugar-sweetened beverages when you are on campus (at university)?

- ☐ Food outlet or store run by the USU (eg. Courtyard; Manning; Hermann's; Laneway; other outlet)
- ☐ Non-USU food outlet or store (eg. Subway; Ralph's; Taste)
- ☐ Other campus food outlet or store (eg. Cumberland; Conservatorium of Music; SCA)
- ☐ Vending machine
- ☐ I don't purchase sugar-sweetened beverages on campus
- ☐ Other (please specify)

____

* 16. Where do you usually purchase sugar-sweetened beverages off campus (outside of the university)?

- ☐ Food outlet (eg. cafe or restaurant; fast-food outlet)
- ☐ Convenience store
- ☐ Supermarket
- ☐ Petrol station
- ☐ Vending machine (eg. train station)
- ☐ I don't purchase sugar-sweetened beverages off campus
- ☐ Other (please specify)

____

Sugar-Sweetened Beverages and their Sale and Promotion in a University

7. Your Opinions about Sugar-Sweetened Beverages

These questions are about the health impacts of consuming sugar-sweetened beverages.

* 17. Do you believe that consumption of sugar-sweetened beverages increases the risk of ill health?

- ◯ Yes
- ◯ No
- ◯ Not sure

* 18. In your opinion, how often do you think people need to drink sugar-sweetened beverages for it to cause ill health?

- ◯ 1 or 2 times per month
- ◯ 1 or 2 times per week
- ◯ 1 time per day
- ◯ 2 times per day
- ◯ 3 times per day
- ◯ 4 times per day
- ◯ I don't believe it causes ill health.

* 19. In your opinion, how often could people drink a 600mL bottle of sugar-sweetened beverage as part of a healthy diet? A 600mL bottle is the most common size of drink available on campus.

- ◯ Every day
- ◯ A few times per week
- ◯ Once or twice per week
- ◯ A few times per month
- ◯ A few times per year
- ◯ I don't think people should drink sugar-sweetened beverages at all.

20. Do you believe that consuming sugar-sweetened beverages increases the risk of developing the following conditions? Tick all that apply.

- [ ] Obesity and overweight
- [ ] Type 2 Diabetes
- [ ] Cardiovascular disease (eg. heart attack)
- [ ] Metabolic syndrome and other related diseases
- [ ] Stroke

Other (please specify)

* 21.

Rank the below activities in the order of 1 to 10, where you believe that 1 is *'not at all damaging to a person's health'* and 10 is *'extremely damaging to a person's health'*. You must provide a different rank for each option ie. each option must be ranked with a number from 1 to 10. You cannot use the same number more than once.

- Smoking 1 pack of cigarettes every week
- Drinking 1 can (375mL) of Coca-Cola a day
- Drinking 4 schooners (425mL) of full strength beer a day
- Eating a McDonalds Big Mac once a week
- Drinking 1 bottle (600mL) of Powerade/Gatorade after exercise three times a week
- Drinking 1 Litre (1000mL) of Diet Coke every week
- Eating bacon every day for breakfast
- Drinking 1 bottle of red or white wine over the weekend (2 days)
- Smoking an occasional cigarette once every month
- Drinking 1 mini bottle (250mL) of flavoured milk a day

* 22. I am trying to cut down on drinking sugar-sweetened beverages.

- ◯ Strongly agree
- ◯ Agree
- ◯ Neither agree nor disagree
- ◯ Disagree
- ◯ Strongly disagree
- ◯ I do not drink sugar-sweetened beverages.

* 23. I would consider choosing a diet or zero sugar-sweetened beverage for health reasons.

- ◯ Strongly agree
- ◯ Agree
- ◯ Neither agree nor disagree
- ◯ Disagree
- ◯ Strongly disagree
- ◯ I do not drink diet or zero sugar-sweetened beverages.

8. Your Opinions about The University of Sydney

These questions are about your opinions regarding the University of Sydney and the sale and promotion of particular products on campus.

* 24. I believe the University of Sydney should promote the health of its students and staff.

- Strongly agree
- Agree
- Neither agree nor disagree
- Disagree
- Strongly disagree

* 25. I believe the University of Sydney should regulate the sale of unhealthy products on its campuses.

- Strongly agree
- Agree
- Neither agree nor disagree
- Disagree
- Strongly disagree

* 26. I believe the University of Sydney should regulate the promotion of unhealthy products on its campuses.

- Strongly agree
- Agree
- Neither agree nor disagree
- Disagree
- Strongly disagree

* 27. I believe the University of Sydney should promote healthier products to its staff and students.

- Strongly agree
- Agree
- Neither agree nor disagree
- Disagree
- Strongly disagree

* 28. I believe there is too much promotion of unhealthy products at the University of Sydney.

- Strongly agree
- Agree
- Neither agree nor disagree
- Disagree
- Strongly disagree

* 29. I believe that products such as sugar-sweetened beverages are harmful for the environment. Eg. In terms of packaging; food miles; carbon dioxide emissions; manufacturing process.

- Strongly agree
- Agree
- Neither agree nor disagree
- Disagree
- Strongly disagree

Sugar-Sweetened Beverages and their Sale and Promotion in a University

9. Your Opinions about the Sale and Promotion of Sugar-Sweetened Beverages on Campus

* 30. Please indicate how supportive or unsupportive you would be of the following measures on campus.

	Extremely supportive	Somewhat supportive	Neutral	Somewhat unsupportive	Extremely unsupportive
Remove discounts on sugar-sweetened beverages (including Access card discounts)	○	○	○	○	○
Increase the price of sugar-sweetened beverages and reinvest the money generated into healthy initiatives on campus (such as a bicycle loan scheme or cheaper healthy food options)	○	○	○	○	○
Remove sugar-sweetened beverages from display (eg. Having them under counter in food outlets)	○	○	○	○	○
Remove the sponsorship and promotion of sugar-sweetened beverages on campus (eg. During O Week and at student events; sporting teams and activities)	○	○	○	○	○
Remove sugar-sweetened beverages from vending machines	○	○	○	○	○
Replace sugar-sweetened beverages with diet or zero sugar-sweetened beverages	○	○	○	○	○
Replace sugar-sweetened beverages with water products	○	○	○	○	○
Encourage healthier beverage options to be placed at eye level in fridges and vending machines	○	○	○	○	○

	Extremely supportive	Somewhat supportive	Neutral	Somewhat unsupportive	Extremely unsupportive
Limit the serving size of sugar-sweetened beverages to less than 250mL in all outlets and machines on campus	○	○	○	○	○
Add nutritional information to all fridges and vending machines on campus	○	○	○	○	○
Run a social marketing campaign to educate staff and students about the health impact of sugar-sweetened beverages	○	○	○	○	○
Increase the price of sugar-sweetened beverages	○	○	○	○	○
Lower the price of water and diet or zero sugar-sweetened beverages	○	○	○	○	○
Increase access to free tap/drinking water on campus	○	○	○	○	○
Remove sugar-sweetened beverages from all campus outlets and vending machines	○	○	○	○	○
Provide access to sugar-sweetened beverages only in campus bars as an alternative to alcohol	○	○	○	○	○

31. Do you have any further comments or suggestions to make regarding sugar sweetened beverages on campus?

Sugar-Sweetened Beverages and their Sale and Promotion in a University

10. Further Studies and Prize Draw

* 32. Do you wish to be contacted regarding future studies in this area of research? You will be asked to provide your University email address in the next question.

- ◯ Yes, I wish to be contacted regarding future studies.
- ◯ No, I do not wish to be contacted once I have participated in this study.

33. Please enter your University email address. If you are a student, this is in the format 'abcd1234@uni.sydney.edu.au'. If you are a staff member, this is in the format 'firstname.lastname@sydney.edu.au'.

Your email address is required in order to be eligible to enter the prize draw for one of five $50 Coles-Myer vouchers and to contact you regarding future studies in this area of research (if you have consented to this).

Please note that this information will be held securely in line with the University of Sydney's Human Ethics Committee rules and procedures regarding personal information.

Thank you for participating in this survey. Once you click 'submit' at the bottom of this page, you will not be able to go back and change your answers.

The results of this survey will be published and disseminated in the University of Sydney and more broadly in the community.
