# Supplementary material for: ‘The university should promote health, but not enforce it’: opinions and attitudes about the regulation of sugar-sweetened beverages in a university setting
Source: BMC Public Health. 2017 Aug 1;18:76. doi: 10.1186/s12889-017-4626-8 (PMC5540300; doi:10.1186/s12889-017-4626-8)
Supplement: Supplementary file 2 — Thematic category descriptors for study qualitative data. (DOCX 20 kb) [file 12889_2017_4626_MOESM2_ESM.docx]

**Appendix B**

| **Category** | **Description of category** | **Number of times appeared in responses** |
| --- | --- | --- |
| Adults are free to make their own decisions or choices | Statements that express the belief or view that adults should be free to make their own choices about what they purchase, eat or drink on campus. This includes statements such as: be able to make informed choices; the university being a place of highly educated people who can make appropriate choices; the concept of over regulation; personal or individual responsibility. | 111 |
| Nanny state | Statements that specifically used the term 'nanny state', a term which has come to refer to public health interventions which are perceived to infringe on the rights of adult individuals. | 13 |
| Remove concessions or increase price on SSBs | Statements that are supportive of either increasing the price of SSBs or removing the discount/concessions in place that make these products cheaper for staff and students. | 8 |
| Make healthy options more available (includes more water fountain access) | Statements that are supportive of increasing the availability of healthier beverage options, including increasing access to water fountains on campus. | 111 |
| Against a ban on SSBs / bans don't work | Statements that were not supportive of removing SSBs from outlets and vending machines, or who raised objections as to why such removal/ban wouldn't work. | 71 |
| Education is an acceptable approach | Statements that expressed the belief or view that educating staff and students on health, including beverage choice, is an appropriate and effective approach. | 72 |
| Concerned about corporate influence | Statements that expressed a concern about the influence of corporate beverage companies in determining choice of and access to specific types and brands of beverages. | 8 |
| Reduce unhealthy options and address unhealthy food environment | Statements that expressed a concern about the broader university food environment in that unhealthy food options are too widely available, and healthier foods are not as accessible. | 35 |
| University should promote health | Statements that specifically noted that the university should promote the health of its students and staff. | 11 |
| Enjoy or need SSBs | Statements that included a range of reasons why for SSBs are acceptable products. Reasons included: enjoying SSBs during particular times of the year (eg. During hotter months); requiring SSBs due to hypoglycemic emergencies for students and staff with Type I diabetes; requiring SSBs are particular times of the semester (eg. energy drinks during exam or assessment time). | 38 |
| Ban bottled beverages due to environmental concerns | Statements that expressed concerns about the environmental impact of bottled beverages in terms of production, waste and recycling. | 24 |
| Support ban on SSBs | Statements that expressed a clear preference or support for banning or removing SSBs from campus entirely. | 13 |
| Concerns about diet or artificially sweetened drinks | Statements that expressed health concerns about diet or artificially-sweetened beverages. | 56 |
| Comments on survey or other issues | Statements that were commenting on the survey, or referring to matters not addressed in the survey (eg. Alcohol). | 51 |
| Unclear | Statements that were unclear in meaning or intent. | 6 |
| **TOTAL** | | **617** |
